# Cardiometabolic risk with use of antipsychotics in children and adolescents

**DOI:** 10.1192/j.eurpsy.2026.10393

**Published:** 2026-07-31

**Authors:** M. Højlund

**Affiliations:** 1 University of Southern Denmark, Odense; 2 Department of Psychiatry Aabenraa, Aabenraa, Denmark

## Abstract

**Abstract:**

This presentation is based on a recent nationwide Danish cohort study examining cardiometabolic risk following antipsychotic initiation across age groups. We will use the study as a framework to illustrate why age at treatment initiation matters clinically. The presentation will show that although absolute cardiometabolic event rates are higher in adults, the excess risk attributable to antipsychotics is greatest when treatment is initiated in childhood and adolescence, particularly before age 12. We will discuss the implications for prescribing thresholds, treatment duration, and metabolic monitoring in younger patients.

**Disclosure of Interest:**

M. Højlund Consultant of: Lundbeck, Orion, and Otsuka, Paid Instructor of: Lundbeck, Orion, and Otsuka.

